# Reproductive biology of the sea anemone shrimp *Periclimenes rathbunae* (Caridea, Palaemonidae, Pontoniinae), from the Caribbean coast of Costa Rica

**DOI:** 10.3897/zookeys.457.7380

**Published:** 2014-11-25

**Authors:** Juan Carlos Azofeifa-Solano, Marcelo Elizondo-Coto, Ingo S. Wehrtmann

**Affiliations:** 1 Escuela de Biología, Universidad de Costa Rica, 11501-2060 San José, Costa Rica; 2 Unidad de Investigación Pesquera y Acuicultura (UNIP), Centro de Investigación en Ciencias del Mar y Limnología (CIMAR), Universidad de Costa Rica, 11501-2060 San José, Costa Rica; 3 Museo de Zoología, Escuela de Biología, Universidad de Costa Rica, 11501-2060 San José, Costa Rica

**Keywords:** Coral reefs, fecundity, new record, reproductive output, symbioses

## Abstract

Caridean shrimps are a highly diverse group and many species form symbiotic relationships with different marine invertebrates. *Periclimenes rathbunae* is a brightly colored shrimp that lives predominantly in association with sea anemones. Information about the reproductive ecology of the species is scarce. Therefore, we collected 70 ovigerous females inhabiting the sun sea anemone *Stichodactyla helianthus* in coral reefs from the southern Caribbean coast of Costa Rica. Females produced on average 289 ± 120 embryos. The volume of recently-produced embryos was on average 0.038 mm^3^, and embryo volume increased by 192% during the incubation period. The average embryo mortality during embryogenesis was 24%. The reproductive output was 0.24 ± 0.094, considerably higher than in many other pontoniine shrimps. Females carrying embryos close to hatching showed fully developed ovaries, suggesting consecutive spawning. We assume that the sheltered habitat, living on sea anemones, allows *Periclimenes rathbunae* to allocate more energy in embryo production than most other free-living caridean shrimps. This is the first record of *Periclimenes rathbunae* for Costa Rica.

## Introduction

Caridean shrimps are a highly diverse group within the Decapoda, comprising approximately 3438 currently valid species within 389 genera (De Grave and Fransen 2011). They inhabit a wide range of habitats (Chace 1972, Bauer 1985, Anker 2003, De Grave et al. 2008), and have different niches (Welsh 1975, Bauer 2004, Hultgren and Duffy 2012), mating behaviors (Berg and Sandifer 1984, Thiel and Hinojosa 2003, Bauer and Thiel 2011, Baeza et al. 2013), and reproductive features (Gherardi and Calloni 1993, Bauer 2000, Echeverría-Sáenz and Wehrtmann 2011, Nye et al. 2013). Caridean shrimps have been relatively well studied, mainly due to the fact that many species are valuable fishery resources (Clarke et al. 1991). Other shrimps have been targeted by the aquarium trade as ornamental species, due to their bright colors and display of associative behavior with other marine species (Calado et al. 2003a, Rhyne et al. 2009).

Many studies report on symbiotic relationships between caridean shrimps and other invertebrates, such as sponges, cnidarians, echinoderms, mollusks, crustaceans, and also with fishes (Bruce 1976, Criales and Corredor 1977, Bauer 2004). The symbiotic partner can receive cleaning services (Limbaugh et al. 1961, Criales and Corredor 1977), protection from predators (Smith 1977), burrow access (Karplus 1987), or increased nitrogen concentrations from shrimp excretions (Spotte 1996). On the other hand, caridean symbionts often benefit by protection from predators or feeding on the host tissue (Fautin et al. 1995, Silbiger and Childress 2008), and increased chances for successful reproduction (Kotb and Hartnoll 2002).

The highly diverse genus *Periclimenes* Costa, 1844 comprises approximately 152 species (De Grave and Fransen 2011). Many species of *Periclimenes* are usually associated with different marine invertebrates such as sea anemones, corals, sea stars and sea cucumbers (Bruce 2004). *Periclimenes rathbunae* Schmitt, 1924 is a brightly colored shrimp recorded from Florida (USA), Mexico, Belize, Colombia, Cuba, Turks and Caicos, Tobago, and Curaçao (Chace 1972, Román-Contreras and Martínez-Mayén 2010). The species has been found associated to a variety of different shallow-water sea anemones such as *Bartholomea annulata* (Le Sueur, 1817), *Bunodosoma granuliferum* (Le Sueur, 1817), *Condylactis gigantea* (Weinland, 1860), *Homostichanthus duerdeni* (Carlgren, 1900), *Lebrunia neglecta* Duchassaing & Michelotti, 1860 and *Stichodactyla helianthus* (Ellis, 1768) (Spotte et al. 1991, Hayes and Trimm 2008). However, it has been also collected from the gorgonian *Eunicea tourneforti* Milne Edwards & Haime, 1857 (see Criales 1980) and from dead corals (Chace 1972). Biological studies on *Periclimenes rathbunae* have focused mainly on their ecology and hosts (Spotte et al. 1991, Hayes and Trimm 2008) but information about reproductive features is scarce (Spotte 1997).

Hines (1982, 1988, 1991) studied the reproductive output (RO) of different marine decapods and reported RO values around 10% for a variety of brachyuran crab species. However, decapod species living as commensals (e.g. in bivalves and corals) and with a reduced calcification of the integument can allocate substantially more energy in embryo production (e.g. pea crabs *Zaops ostreus* (Say, 1817) and *Fabia subquadrata* Dana, 1851: Hines 1992; coral gall crab *Hapalocarcinus marsupialis* Stimpson, 1859: Kotb and Hartnoll 2002). Here we studied fecundity and reproductive output of *Periclimenes rathbunae* in order to test the hypothesis that pontoniine shrimps living as symbionts with other invertebrates can invest more energy in embryo production that free-living shrimps but less than decapods living enclosed in other invertebrates.

## Methods

**Figure 1.**
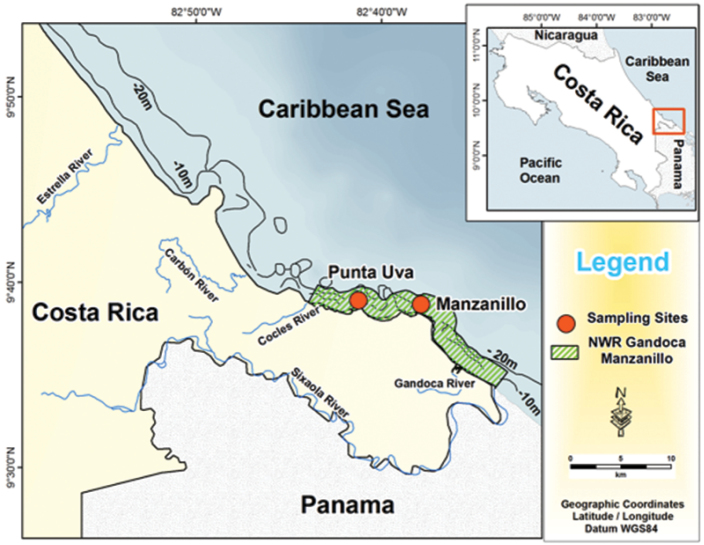
Sampling sites of *Periclimenes rathbunae* visited between 2011 and 2012: Punta Uva and Manzanillo beaches, Gandoca-Manzanillo National Wild-Life Refuge, Caribbean coast of Costa Rica.

Ovigerous females of *Periclimenes rathbunae* were collected during five field trips (September and October 2011, January, June and October 2012) in the Puerto Viejo-Punta Mona coral reef area (Cortés et al. 2010) within the Gandoca-Manzanillo National Wild Life Refuge, at the southern Caribbean coast of Costa Rica (Fig. 1). The sea surface temperature in all sampling months varied between 27 and 30 °C (data provided by MIO-CIMAR: http://www.miocimar.ucr.ac.cr/). The substrate was dominated by algal ridges, with low live coral cover, ranging from 8 to 16%, although this percentage has been increasing during the last decades (Cortés et al. 2010). All specimens were collected between 1-4 m depth, and were associated with the sun sea anemone, *Stichodactyla helianthus* Ellis, 1768. The shrimps were collected by hand during snorkeling dives and placed individually into plastic vials. The collected specimens were stored and preserved in 70% ethanol, and subsequently transported to the laboratory at the Escuela de Biología, Universidad de Costa Rica, in San José. The shrimps were identified according to Chace (1972) and photos provided by Dr Arthur Anker. Six specimens were deposited in the Museo de Zoología of the Universidad de Costa Rica (catalog number MZUCR 3155-01). The material was collected under the sampling permit No. 181-2013 provided by SINAC-MINAET.

The total length (TL, distance between distal part of the eye socket to the distal margin of the telson excluding setae) and carapace length (CL, distance between distal part of the eye socket to the posterior margin of the carapace) were measured with the aid of Leica MS5 stereoscopic microscope equipped with a calibrated ocular micrometer. Linear regression was performed to test the relationship between TL and CL. The entire embryo mass was removed from females and photographed to count the number of embryos, using IMAGE TOOL version 3.00 developed by UTHSCSA. Here we used the term fecundity as the number of embryos carried by the female (Corey and Reid 1991). The female length and the number of embryos were correlated using linear regression analysis. Thirty embryos of each clutch were separated, and length and width of embryos were measured under a Leica CME microscope equipped with a calibrated ocular micrometer. The embryo volume was calculated using the formula for oblate spheroids V = 1/6 (*π* d_1_ × d_2_^2^) where d_1_ is the mayor diameter, and d_2_ is the perpendicular diameter (Turner and Lawrence 1979). The brood mass volume was estimated multiplying total embryo number per female by their respective average embryo volume (Echeverría-Sáenz and Wehrtmann 2011).

The stage of embryo development was assigned following the criteria described by Wehrtmann (1990): Stage I, uniform yolk, no eye pigments observed; Stage II, eye pigments start to develop; Stage III, embryo clearly visible and fully developed. The number of embryos, embryo volume and brood mass volume were compared between developmental stages of the embryos using one-way analysis of variance. Females and brood masses were dried separately at 60 °C for 48 hours, and the dry weight was measured using a Sartorius TE64 analytical balance (± 0.0001) to calculate the reproductive output (RO): dry weight of total brood mass per female divided by dry weight of female without brood mass (Hines 1988, Echeverría-Sáenz and Wehrtmann 2011). The RO was estimated exclusively for females carrying recently-extruded embryos (Stage I).

The stage of ovarian development was determined following the criteria proposed by Bauer (1986): Stage 1, no noticeable development; Stage 2, vitellogenic oocytes distinct but small ovary; Stage 3, ovary filling at least half the space above the cardiac stomach; Stage 4, ovary completely filling the space above the cardiac stomach. The ovarian and embryos development were analyzed to infer the possibility of consecutive spawning (Bauer 1986, 1992).

## Results

A total of 70 ovigerous females of *Periclimenes rathbunae* were analyzed; TL of these specimens was directly proportional to CL (CL = 0.1657 × TL + 0.5497; F = 313.21; DF = 69; P < 0.001; R^2^ = 0.80). Individuals ranged in size from 2.25 to 5.25 mm CL with an average of 3.98 ± 0.77 mm CL. A total of 29 females carried embryos in Stage I, 14 in Stage II, and 27 in Stage III.

### Embryo number

**Figure 2.**
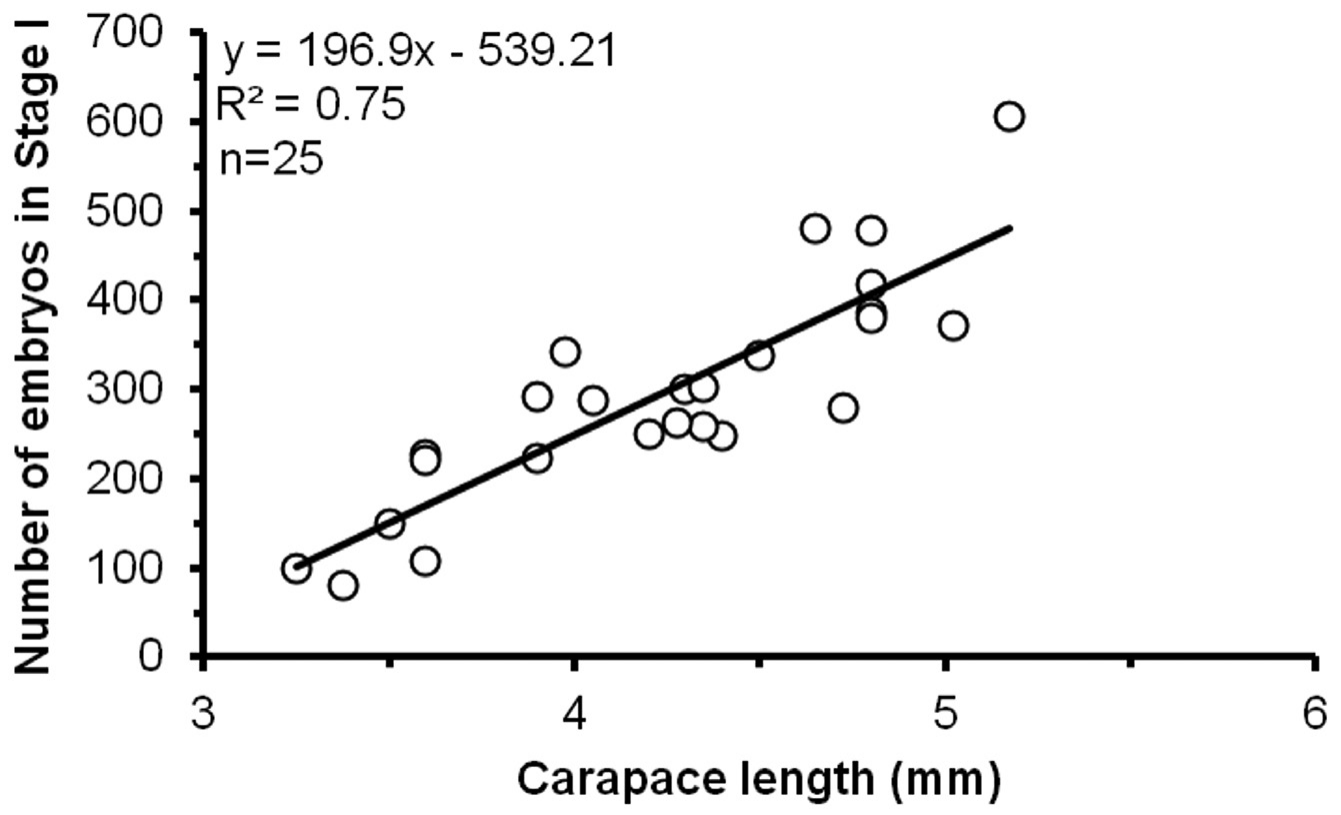
Relation between number of embryos in Stage I and female size of *Periclimenes rathbunae* from the Caribbean coast of Costa Rica, 2011–2012.

The average number of Stage I embryos was 289 ± 120 embryos per female, with a minimum and maximum of 80 and 605, respectively. The number of recently-extruded embryos (Stage I) increased significantly with female size (F = 69.1; DF = 23; P < 0.001; R^2^ = 0.75) (Fig. 2). The embryo number was significantly different between Stage I and III (F = 3.5; DF = 66; P = 0.03) (Table 1), but this difference was due to a significant decrease of embryo numbers from Stage II to Stage III (F = 4.7; DF = 39; P = 0.03). Average embryo number decreased during the incubation period by 24%.

**Table 1.** Number of analyzed specimens, mean ± standard deviation of carapace length (CL), embryo number, embryo volume, and brood mass volume according to the stage of embryo development in females of *Periclimenes rathbunae* from the Caribbean coast of Costa Rica, 2011–2012.

Stage of embryo development	n	CL (mm)	Embryo number	Embryo volume (mm^3^)	Brood mass volume (mm^3^)
I	29	4.2 ± 0.6	289 ± 120	0.038 ± 0.011	10.6 ± 4.6
II	14	4.3 ± 0.5	288 ± 105	0.050 ± 0.013	15.0 ± 6.8
III	27	4.2 ± 0.5	219 ± 90	0.072 ± 0.022	15.1 ± 6.7

### Embryo volume and brood mass volume

Recently-produced embryos (Stage I) had an average volume of 0.038 mm^3^, and those closed to hatching (Stage III) 0.073 mm^3^ (Table 1), representing a volume increase of 192% during the incubation period. The average embryo volume (F = 30.9; DF = 67; P < 0.001) as well the average brood mass volume (F = 4.5; DF = 66; P = 0.01) was statistically different among the three developmental stages (Table 1). Average brood mass volume increased during embryogenesis from 10.6 to 15.1 mm^3^ (Table 1), which represented a 42% increase.

### Reproductive output

**Figure 3.**
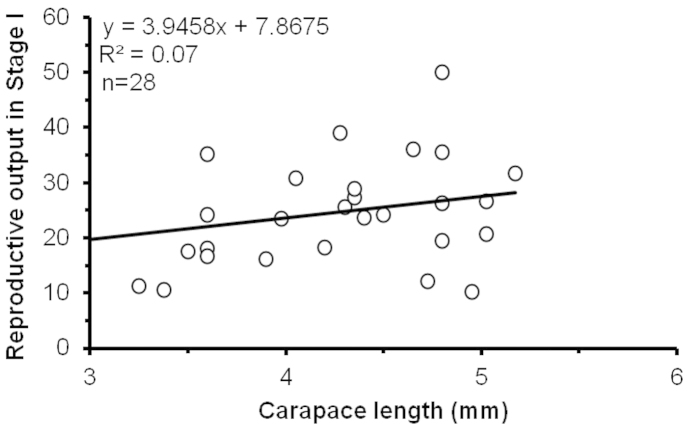
*Periclimenes rathbunae* from the Caribbean coast of Costa Rica, 2011–2012: relation between reproductive output (Stage I) and female size.

The average RO for female *Periclimenes rathbunae* was 0.24 ± 0.094, fluctuating between 0.10 and 0.50. There was no significant correlation between RO and CL of females (F = 2.0; DF = 26; P > 0.05; R^2^ = 0.07) (Fig. 3).

### Reproductive activity

**Figure 4.**
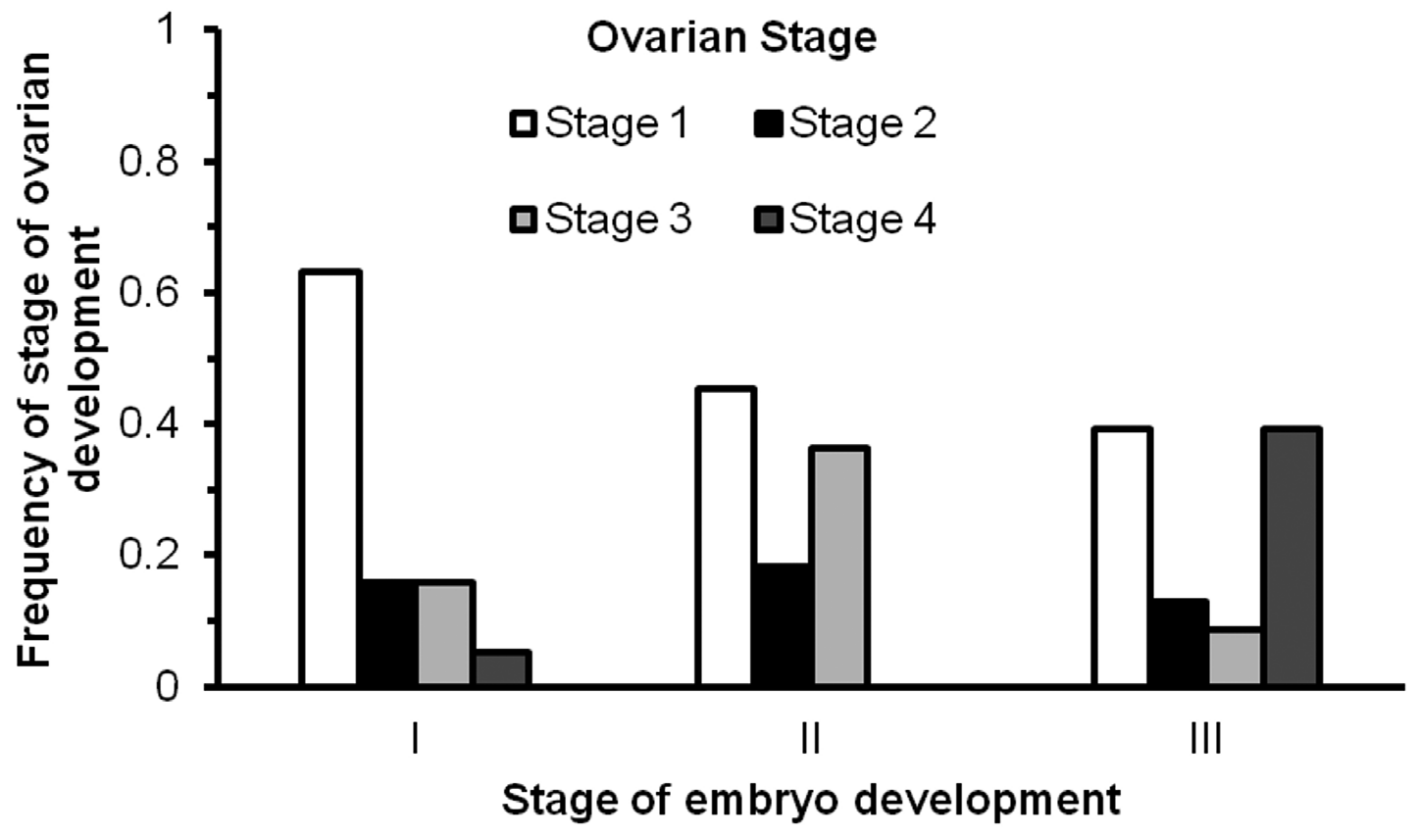
Frequency of stage of ovarian development in relation to the stage of embryo development from females of *Periclimenes rathbunae* from the Caribbean coast of Costa Rica, 2011–2012.

Ovigerous females were collected during all five field trips carried out between September 2011 and October 2012. Early ovarian stages (Stage 1–2) predominated in females carrying recently-extruded embryos (Stage I), while ovaries filled with vitellogenic oocytes (Stage 4) reached its highest occurrence in females with embryos close to hatching (Stage III) (Fig. 4).

## Discussion

This is the first record of *Periclimenes rathbunae* for Costa Rica. Vargas and Wehrtmann (2009) summarized the available information on marine decapod diversity in Costa Rica, but did not mention *Periclimenes rathbunae*; however, they pointed out that the Caribbean coast of Costa Rica was less studied than the Pacific coast of the country. This new record confirms the geographic distribution of the species, ranging from Florida (USA) to Curaçao (Chace 1972, Román-Contreras and Martínez-Mayén 2010).

### Embryo number

The sea anemone shrimp *Periclimenes rathbunae* produces a relatively high number of offspring when compared to other pontoniine species (Table 2): only *Periclimenes ornatus* Bruce, 1969 has been reported to carry more embryos (Omori et al. 1994), but this species reaches also slightly larger sizes. Many studies on caridean shrimps revealed that fecundity is closely related to female size (for tropical palaemonids: Anger and Moreira 1998, Wehrtmann and Lardies 1999, Nazari et al. 2003, da Silva et al. 2004, Lara and Wehrtmann 2009, Meireles et al. 2013), and our data for *Periclimenes rathbunae* confirm this tendency (Fig. 2). We observed also sexual dimorphism in *Periclimenes rathbunae* with females reaching larger sizes than males (JC Azofeifa-Solano et al., unpubl data), a phenomenon well-known in many caridean shrimps (Bauer 2004), and also reported for *Periclimenes ornatus* by Omori et al. (1994) who found that males attained smaller sizes, but had larger chelae than females.

**Table 2.** Minimum and maximum female carapace length (CL), embryo number in Stage I, mean embryo volume and reproductive output (RO) in Stage I, habitat and study site of seven pontoniine species; NA = no data available; * = total length. ** = Value recalculated by the authors of the present study (see Discussion).

Species	CL (mm)	Embryo number	Embryo volume (mm^3^)	RO (%)	Habitat	Study site	Reference
*Periclimenes ornatus* Bruce, 1969	3.0–6.0	10–1000	0.49 (0.06**)	NA	Sea anemone	Shikoku Island, Japan	Omori et al. (1994)
*Ancylomenes pedersoni* (Chace, 1958) Reported as *Periclimenes anthophilus*	NA	33–80	0.05	NA	Sea anemone	Bermuda	Spotte (1999)
*Ancylomenes pedersoni* (Chace, 1958) Reported as *Periclimenes pedersoni*	NA	78–221	0.11	NA	Sea anemone	Bahamas	Spotte (1999)
*Periclimenes pandionis* Holthuis, 1951	2.84–4.0	67–259	0.05	NA	Coral reef	Indian River, Florida	Corey and Reid (1991)
*Periclimenes patae* Heard & Spotte, 1991	3.3–4.2	10–35	NA	NA	Gorgonian	Turks and Caicos and Florida Keys	Heard and Spotte (1991)
*Periclimenes yucatanicus* (Ives, 1891)	3.52–5.73	12–333	NA	NA	Sea anemone	West Indies and south Florida	Spotte (1997)
*Phycomenes siankaanensis* (Martinez-Mayén & Román-Contreras, 2006)	1.91–3.2	23–141	0.056	NA	Sea grass meadow	Quintana Roo, Yucatan Peninsula, Mexico	Martínez-Mayén and Román-Contreras (2009)
*Periclimenes rathbunae* Schmitt, 1924	2.25–5.25 (12.3–22.6*)	80–605	0.038	24.0 ± 0.09	Sea anemone	Limón, Costa Rica	Present study

### Embryo volume and brood mass volume

The average embryo volume of *Periclimenes rathbunae* is in the range of most values reported for other pontoniine species (Table 2). However, *Periclimenes ornatus* in southwest Japan produces considerably larger embryos with a maximum volume of 0.49 mm^3^ (Omori et al. 1994). However, these authors did not explain how they calculated the embryo volume. Using the average values for embryo length and width provided by Omori et al. (1994), and applying the same equation as used in the present study (Turner and Lawrence 1979), average embryo volume of *Periclimenes ornatus* would be 0.06 mm^3^, which is in the range reported for other pontoniine species, including *Periclimenes rathbunae* (Table 2). Both species, *Periclimenes rathbunae* and *Periclimenes ornatus*, share similar size ranges and live associated with sea anemones in tropical and subtropical waters (Chace 1972, Omori et al. 1994). Most species of pontoniine shrimps producing these relatively small embryos, including *Periclimenes rathbunae*, probably have an extended larval development. There is still a great lack of knowledge concerning the complete larval development of most of the pontoniine species; however, dos Santos et al. (2004) cultivated *Periclimenes sagittifer* Norman, 1861 in the laboratory and described eight zoeal stages, corroborating an extended larval period.

*Periclimenes rathbunae* lost during the incubation period on average 24% of the initially-produced embryos, while embryo volume increased by 192%. Brood loss in *Periclimenes rathbunae* was similar to that reported for other palaemonid freshwater shrimps: approximately 23% in *Palaemon pandaliformis* (Stimpson, 1871) and *Macrobrachium acanthurus* (Wiegmann, 1836) (Kuris 1991, Anger and Moreira 1998) and was slightly higher than the 17.2% of brood loss observed in the marine palaemonid *Palaemon gravieri* (Yu, 1930) (see Kim and Hong 2004). Furthermore, it was higher than the brood loss reported for the sponge-dwelling snapping shrimp *Synalpheus yano* (Ríos & Duffy, 2007) from Panama (Hernáez et al. 2010). The volume increase of caridean shrimp embryos during the incubation period ranges from 3.9 to 155.9% (Corey and Reid 1991); thus the observed value for *Periclimenes rathbunae* is considerably higher than this range. The combination of relatively low brood loss and substantial embryo volume increase during embryogenesis in *Periclimenes rathbunae* suggests that the physical space available for embryo attachment is sufficient to accommodate and maintain the clutch until hatching. The association with the sea anemone and efficient parental care during the incubation period may provide favorable conditions for the embryo development, and thus reducing brood loss during the embryogenesis. Such an interpretation is in agreement with similar observations in *Synalpheus yano*, a pair-living and sponge-dwelling alpheid species from tropical waters (Hernáez et al. 2010).

### Reproductive output

The RO is a widely used instrument to document and compare inter- and intra-specifically energy allocation in offspring production of decapod crustaceans (Clarke et al. 1991, Hines 1991, Lardies and Wehrtmann 1996, Anger and Moreira 1998, Terossi et al. 2010, Echeverría-Sáenz and Wehrtmann 2011). To our best knowledge, here we report the first RO value for any pontoniine species (Table 2). The RO of *Periclimenes rathbunae* females (0.24) was higher than that reported for some free-living marine shrimps: 14.4 ± 2.5% for *Palaemon northropi* (Rankin, 1898) (see Anger and Moreira 1998) and 17.8 ± 6.0% for *Heterocarpus vicarius* Faxon, 1893 (see Echeverría-Sáenz and Wehrtmann 2011); and freshwater shrimps: 18.6 ± 3.0% for *Palaemon pandaliformis* (Stimpson, 1871) (see Anger and Moreira 1998), 19.1 ± 4.5% for *Macrobrachium acanthurus* (Wiegmann, 1836) (see Anger and Moreira 1998), 21.7 ± 6.6% for *Macrobrachium olfersii* (Wiegmann, 1836) (see Anger and Moreira 1998), and 3.6 ± 1.9% for *Atya scabra* (Leach, 1816) (see Herrera-Correal et al. 2013). These results suggest that marine caridean shrimps species living in association with other invertebrates are able to invest on average more energy in brood production than free-living species. Nevertheless, we suggest analyzing the RO within the subfamily Pontoniinae between free living and symbiont species in order to test if a sheltered habitat is related to an increase in RO in this diverse group of shrimps.

The reduction of the calcification of the exoskeleton results in a minimized dry weight of the decapod species, thus increasing its relative brood weight (Hines 1992; Kotb and Hartnoll 2002). The symbiont *Hapalocarcinus marsupialis*, a species which provokes gall development in its host coral has a reproductive investment per brood of 59% (Kotb and Hartnoll 2002), and this value is still lower than those published for symbiotic decapods, such as the pea crabs *Zaops ostreus* (66%) and *Fabia subquadrata* (97%) (Hines 1992). While the coral gall crab and the pea crabs live protected within galls of corals and bivalves, respectively, species like *Periclimenes rathbunae* are associates on other invertebrates, more exposed to predation and wave action than the above-mentioned crab species. This may explain the relatively high RO value in *Periclimenes rathbunae* compared to free-living decapods, but substantially lower RO-value when compared to symbiotic decapods living enclosed within their host. Additional studies with decapod species living in association with other invertebrates are needed to substantiate the hypothesis of increasing energy allocation in brood production with increasing degree of protection provided by the host.

### Reproductive activity

Relatively elevated and stable temperatures in tropical seas may allow year-round reproduction of marine decapods (Bauer 1986, 1992). Our results concerning the relation between the state of ovarian development and stage of embryo development as well as the fact that ovigerous females of *Periclimenes rathbunae* were encountered throughout the sampling period confirms that this species has continuous reproduction (Mossolin and Bueno 2002). Bauer (1992) studied reproductive patterns in different caridean shrimps, including *Cuapetes americanus* (Kingsley, 1878) associated with sea grass meadows in Puerto Rico; his results revealed that all of these caridean species go through continuous cycles of ovarian maturity and spawning until they die. Our results corroborate the generalized pattern of continuous reproduction in tropical shallow-water shrimps and confirm *Periclimenes rathbunae* as an iteroparous species.

In recent years, the pressure on ornamental species has increased, and this includes also decapod species, which are highly popular among aquarium hobbyists (Calado et al. 2003a). As far as we know, *Periclimenes rathbunae* has not been harvested yet at the Caribbean coast of Costa Rica; however, its eye-catching color pattern and the fact that the shrimp lives in association with sea anemones makes it a potential candidate for aquarium hobbyists, just as numerous other pontoniine species (see Calado et al. 2003a). In order to minimize possible negative impacts caused by the harvest of wild marine species such as *Periclimenes rathbunae* (see Wood 2001), additional information on larval development is needed to cultivate the early life stages under controlled laboratory conditions (Calado et al. 2003b). Moreover, many other aspects of the ecology of *Periclimenes rathbunae* remain to be studied, e.g., mating behavior, recruitment, settlement on the host species as well as other details about the association of the shrimp with its host.

## Acknowledgements

We would like to thank all volunteers who assisted this study, especially Moisés Pérez and Wagner Cháves. We are very grateful to Raymond Bauer for his recommendations and encouraging comments regarding this study. We are grateful to Raquel Romero who prepared the map of the sampling area. Special thanks go to Arthur Anker who provided photos to facilitate the *in situ* identification of the shrimp. We are very thankful to Rita Vargas who corroborated the identification of species, and to Carolina Salas for her help to deposit the specimens in the Museo de Zoología of the Universidad de Costa Rica. We sincerely appreciated the valuable comments of two anonymous reviewers, which further improved the quality of the manuscript.

## References

[B1] Anger K Moreira GS (1998) Morphometric and reproductive traits of tropical caridean shrimps. Journal of Crustacean Biology 18(4): 823–838. doi: 10.2307/1549156

[B2] Anker A (2003) Alpheid shrimps from the mangroves and mudflats of Singapore. Part I. Genera *Salmoneus*, *Athanas* and *Potamalpheops*, with the description of two new species (Crustacea: Decapoda: Caridea). The Raffles Bulletin of Zoology 51(2): 283–314.

[B3] Baeza JA Ritson-Williams R Fuentes MS (2013) Sexual and mating system in a caridean shrimp symbiotic with the winged pearl oyster in the Coral Triangle. Journal of Zoology 289(3): 172–181. doi: 10.1111/j.1469-7998.2012.00974.x

[B4] Bauer RT (1985) Diel and seasonal variation in species composition and abundance of caridean shrimps (Crustacea, Decapoda) from sea grass meadows on the north coast of Puerto Rico. Bulletin of Marine Science 36: 150–162.

[B5] Bauer RT (1986) Sex change and life history pattern in the shrimp *Thor manningi* (Decapoda: Caridea): a novel case of partial protandric hermaphroditism. The Biological Bulletin 170: 11–31. doi: 10.2307/1541377

[B6] Bauer RT (1992) Testing generalization about latitudinal variation in reproduction and recruitment patterns with sicyoniid and caridean shrimp species. Invertebrate Reproduction and Development 22(1–3): 193–202. doi: 10.1080/07924259.1992.9672272

[B7] Bauer RT (2000) Simultaneous hermaphroditism in caridean shrimps: a unique and puzzling sexual system in the Decapoda. Journal of Crustacean Biology 20(2): 116–128.

[B8] Bauer RT (2004) Remarkable Shrimps: Adaptations and Natural History of the Carideans. University of Oklahoma Press, Oklahoma, USA, 282 pp.

[B9] Bauer RT Thiel M (2011) First description of a pure-research mating system and protandry in the shrimp *Rhynchocinetes uritai* (Decapoda: Caridea). Journal of Crustacean Biology 31(2): 286–295. doi: 10.1651/10-3378.1

[B10] Berg ABV Sandifer PA (1984) Mating behavior of the sea grass shrimp *Palaemonetes pugio* Holthuis (Decapoda, Caridea). Journal of Crustacean Biology 4(3): 417–424. doi: 10.2307/1548041

[B11] Bruce AJ (1976) Shrimps and prawns of coral reef, with special reference to commensalism. In: Jones O Endean R (Eds) Biology and Geology of Coral Reefs, Vol III, Biology 2 Academic Press, New York, USA, 38–94.

[B12] Bruce AJ (2004) A partial revision of the genus *Periclimenes* Costa, 1884 (Crustacea: Decapoda: Palaemonidae). Zootaxa 582: 1–26.

[B13] Calado R Lin J Rhyne AL Araújo R Narciso L (2003a) Marine ornamental decapods: popular, pricey and poorly studied. Journal of Crustacean Society 23(4): 963–973. doi: 10.1651/C-2409

[B14] Calado R Narciso L Morais S Rhyne AL Lin J (2003b) A rearing system for the culture of ornamental decapod crustacean larvae. Aquaculture 218: 329–339. doi: 10.1016/S0044-8486(02)00583-5

[B15] Chace FA Jr (1972) The shrimps of the Smithsonian-Bredin Caribbean expeditions with a summary of the West Indian shallow-water species (Crustacea: Decapoda: Natantia). Smithsonian Institution Press, Washington, USA, 179 pp.

[B16] Clarke A Hopkins CCE Nilssen EM (1991) Egg size and reproductive output in the deep-water prawn *Pandalus borealis* Krøyer, 1838. Functional Ecology 5(6): 724–730. doi: 10.2307/2389534

[B17] Corey S Reid DM (1991) Comparative fecundity of decapod crustaceans I. The fecundity of thirty-three species of nine families of caridean shrimp. Crustaceana 60(3): 270–296. doi: 10.1163/156854091X00056

[B18] Cortés J Jiménez CE Fonseca AC Alvarado JJ (2010) Status and conservation of coral reefs in Costa Rica. Revista de Biología Tropical 58: 33–50. 20873039 10.15517/rbt.v58i1.20022

[B19] Criales MM (1980) Commensal caridean shrimps of Octocorallia and Antipatharia in Curacao and Bonaire. Studies on the Fauna of Curacao and other Caribbean Island 61: 68–85.

[B20] Criales MM Corredor L (1977) Aspectos etológicos y ecológicos de camarones limpiadores de peces (Natantia: Palaemonidae, Hippolytidae, Stenopodidae). Anales del Instituto de Investigaciones Marinas, Punta Betín 9: 141–156.

[B21] Da Silva RR Sampaio CMS Santos JA (2004) Fecundity and fertility of *Macrobrachium amazonicum* (Crustacea, Palaemonidae). Brazilian Journal of Biology 64: 489–500. doi: 10.1590/S1519-69842004000300012 10.1590/s1519-69842004000300012 15622846

[B22] De Grave S Fransen CHJM (2011) Carideorum catalogus: the recent species of the dendrobranchiate, stenopodidean, procarididean and caridean shrimps (Crustacea: Decapoda). Zoologische Mededelingen, Leiden 85(9): 195–589.

[B23] De Grave S Cai Y Anker A (2008) Global diversity of shrimps (Crustacea: Decapoda: Caridea) in freshwater. Freshwater Animal Diversity Assessment 595: 287–293. doi: 10.1007/978-1-4020-8259-7_31

[B24] Dos Santos A Calado R Bartilotti C Narciso L (2004) The larval development of the partner shrimp *Periclimenes sagittifer* (Norman, 1861) (Decapoda: Caridea: Palaemonidae: Pontoniinae) described from laboratory-reared material, with a note on chemical settlement cues. Helgoland Marine Research 58: 129–139. doi: 10.1007/s10152-004-0178-2

[B25] Echeverría-Sáenz S Wehrtmann IS (2011) Egg production of the commercially exploited deepwater shrimp, *Heterocarpus vicarius* (Decapoda: Pandalidae), Pacific Costa Rica. Journal of Crustacean Biology 31: 434–440. doi: 10.1651/10-3400.1

[B26] Fautin DG Guo CC Hwang JS (1995) Cost and benefits of the symbiosis between the anemone shrimp *Periclimenes brevicarpalis* and its host *Entacmaea quadricolor*. Marine Ecology Progress Series 129: 77–84. doi: 10.3354/meps129077

[B27] Gherardi F Calloni C (1993) Protandrus hermaphroditism in the tropical shrimp *Athanas indicus* (Decapoda: Caridea), a symbiont of sea urchins. Journal of Crustacean Biology 13(4): 675–689. doi: 10.2307/1549098

[B28] Hayes FE Trimm NA (2008) Distributional ecology of the anemone shrimp *Periclimenes rathbunae* associating with the sea anemone *Stichodactyla helianthus* at Tobago, West Indies. Nauplius 16: 73–77.

[B29] Heard RW Spotte S (1991) Pontoniinae shrimps (Decapoda: Caridea: Palaemonidae) of the northwest Atlantic. II. *Periclimenes patae*, new species, a gorgonian associate from shallow reef areas off the Turks and Caicos Islands and Florida Keys. Proceeding of the Biological Society of Washington 104: 40–48.

[B30] Hernáez P Martínez-Guerrero B Anker A Wehrtmann IS (2010) Fecundity and effects of bopyrid infestation on egg production in the Caribbean sponge-dwelling snapping shrimp *Synalpheus yano* (Decapoda: Alpheidae). Journal of the Marine Biological Association of the United Kingdom 90: 691–698. doi: 10.1017/S0025315409991093

[B31] Herrera-Correal J Mossolin EC Wehrtmann IS Mantelatto FL (2013) Reproductive aspects of the caridean shrimp *Atya scabra* (Leach, 1815) (Decapoda: Atyidae) in São Sebastião Island, southwestern Atlantic, Brazil. Latin American Journal of Aquatic Research 41(4): 676–684. doi: 10.3856/vol41-issue4-fulltext-4

[B32] Hines AH (1982) Allometric constraints and variables of reproductive effort in brachyuran crabs. Marine Biology 69: 309–320. doi: 10.1007/BF00397496

[B33] Hines AH (1988) Fecundity and reproductive output in two species of deep sea crabs, *Greyson fenerri* and *G. quinquedens* (Decapoda, Brachyura). Journal of Crustacean Biology 8: 557–562. doi: 10.2307/1548692

[B34] Hines AH (1991) Fecundity and reproductive output in nine species of *Cancer* crabs (Crustacea, Brachyura, Cancridae). Canadian Journal of Fisheries and Aquatic Sciences 48: 267–275. doi: 10.1139/f91-037

[B35] Hines AH (1992) Constraint of reproductive output in brachyuran crabs: pinnotherids test the rule. American Zoologist 32: 503–511.

[B36] Hultgren KM Duffy JE (2012) Phylogenetic community ecology and the role of social dominance in sponge-dwelling shrimp. Ecology Letters 15(7): 704–713. doi: 10.1111/j.1461-0248.2012.01788.x 22548770 10.1111/j.1461-0248.2012.01788.x

[B37] Karplus I (1987) The association between gobiid fishes and burrowing alpheid shrimps. Oceanography and Marine Biology-Annual Review 25: 507–562.

[B38] Kim S Hong S (2004) Reproductive biology of *Palaemon gravieri* (Decapoda: Caridea: Palaemonidae). Journal of Crustacean Biology 24: 121–130. doi: 10.1651/C-2369

[B39] Kotb MMA Hartnoll RG (2002) Aspects of the growth and reproduction of the coral gall crab *Hapalocarcinus marsupialis*. Journal of Crustacean Biology 22(3): 558–566. doi: 10.1163/20021975-99990268

[B40] Kuris AM (1991) A review of patterns and causes of crustacean brood mortality. In: Wenner A Kuris A (Eds) Crustacean Egg Production. AA Balkema, Rotterdam Crustacean Issues 7: 117–141.

[B41] Lara R Wehrtmann IS (2009) Reproductive biology of the freshwater shrimp *Macrobrachium carcinus* (L.) (Decapoda: Palaemonidae) from Costa Rica, Central America. Journal of Crustacean Biology 29: 343–349. doi: 10.1651/08-3109.1

[B42] Lardies MA Wehrtmann IS (1996) Aspects of the reproductive biology of *Petrolisthes laevigatus* (Guérin, 1835) (Decapoda, Anomura, Porcellanidae). Part I: Reproductive output and chemical composition of eggs during embryonic development. Archive of Fishery and Marine Research 43: 121–135.

[B43] Limbaugh C Pederson H Chace FA Jr (1961) Shrimps that clean fishes. Bulletin of Marine Science of the Gulf and Caribbean 11: 237–257.

[B44] Martínez-Mayén M Román-Contreras R (2009) Reproduction of *Periclimenes siankaaensis* (Decapoda, Caridea, Palaemonidae) in Bahía de la Ascensión, Quintana Roo, Mexico. Crustaceana 82: 27–37. doi: 10.1163/156854008X389599

[B45] Meireles AL Valenti WC Mantelatto FL (2013) Reproductive variability of the Amazon River prawn, *Macrobrachium amazonicum* (Caridea, Palaemonidae): influence of life cycle on egg production. Latin American Journal of Aquatic Research 41: 718–731. doi: 10.3856/vol41-issue4-fulltext-8

[B46] Mossolin EC Bueno SLS (2002) Reproductive biology of *Macrobrachium olfersi* (Decapoda, Palaemonidae) in São Sebastião, Brazil. Journal of Crustacean Biology 22(2): 367–376. doi: 10.1163/20021975-99990244

[B47] Nazari EM Simões-Costa MS Müller YMR Ammar D Dias M (2003) Comparisons of fecundity, egg size, and egg mass volume of the freshwater prawns *Macrobrachium potiuna* and *Macrobrachium olfersi* (Decapoda, Palaemonidae). Journal of Crustacean Biology 23: 862–868. doi: 10.1651/C-2387

[B48] Nye V Copley JT Tyler PA (2013) Spatial variation in the population structure and reproductive biology of *Rimicaris hybisae* (Caridea: Alvinocarididae) at hydrothermal vents on the Mid-Cayman Spreading Centre. PLoS ONE 8(3): . doi: 10.1371/journal.pone.0060319 10.1371/journal.pone.0060319 PMC3612092 23555955

[B49] Omori K Yanagisawa Y Hori N (1994) Life history of the caridean shrimp *Periclimenes ornatus* Bruce associated with a sea anemone in the southwest Japan. Journal of Crustacean Biology 14: 132–145. doi: 10.2307/1549060

[B50] Rhyne A Rotjan R Bruckner A Tlusty M (2009) Crawling to collapse: ecologically unsound ornamental invertebrate fisheries. PLoS ONE 4: . doi: 10.1371/journal.pone.0008413 10.1371/journal.pone.0008413 PMC2793429 20027312

[B51] Román-Contreras R Martínez-Mayén M (2010) Palaemonidae (Crustacea: Decapoda: Caridea) from the shallow waters from Quintana Roo, Mexican Caribbean coast. Revista Mexicana de Biodiversidad 81: 43–51.

[B52] Silbiger NJ Childress MJ (2008) Interspecific variation in anemone shrimp distribution and host selection in the Florida Keys (USA): implications for marine conservation. Bulletin of Marine Science 83: 329–345.

[B53] Smith WL (1977) Beneficial behavior of a symbiotic shrimp to its host anemone. Bulletin of Marine Sciences 27: 343–346.

[B54] Spotte S (1996) Supply of regenerated nitrogen to sea anemones by their symbiotic shrimp. Journal of Experimental Marine Biology and Ecology 198: 27–36. doi: 10.1016/0022-0981(95)00169-7

[B55] Spotte S (1997) Sexual and regional variation in the morphology of the spotted anemone shrimp *Periclimenes yucatanicus* (Decapoda: Caridea: Palaemonidae). Journal of Crustacean Biology 17(3): 389–397. doi: 10.2307/1549433

[B56] Spotte S (1999) Possible synonymy of the western Atlantic anemone shrimps *Periclimenes pedersoni* and *P. anthophilus* based on morphology. Bulletin of Marine Science 65: 407–417.

[B57] Spotte S Heard RW Bubucis PM Manstan RR McLelland JA (1991) Pattern and coloration of *Periclimenes rathbunae* from the Turks and Caicos Islands, with comments on host associations in other anemones shrimps of the West Indies and Bermuda. Gulf Research Reports 8: 301–311.

[B58] Terossi M Wehrtmann IS Mantelatto FL (2010) Interpopulation comparison of reproduction of the Atlantic shrimp *Hippolyte obliquimanus* (Caridea: Hippolytidae). Journal of Crustacean Biology 30(4): 571–579. doi: 10.1651/09-3233.1

[B59] Thiel M Hinojosa IA (2003) Mating behavior of female rock shrimp *Rhynchocinetes typus* (Decapoda: Caridea) - indication for convenience polyandry and cryptic female choice. Behavior Ecology and Sociobiology 55(2): 113–121. doi: 10.1007/s00265-003-0677-1

[B60] Turner RL Lawrence JM (1979) Volume and composition of echinoderm eggs: implications for the use of egg size in life-history models. In: Stancyk SE (Ed.) Reproductive Ecology of Marine Invertebrates. University of South Carolina Press, Columbia, USA, 25–40.

[B61] Vargas R Wehrtmann IS (2009) Decapod crustaceans. In: Wehrtmann IS Cortés J (Eds) Marine Biodiversity of Costa Rica, Central America. Springer Science + Business Media BV, Berlin, Germany, 209–228. doi: 10.1007/978-1-4020-8278-8_19

[B62] Wehrtmann IS (1990) Distribution and reproduction of *Ambidexter panamense* and *Palaemonetes schmitti* in the Pacific Costa Rica (Crustacea, Decapoda). Revista de Biología Tropical 38(2A): 327–329.

[B63] Wehrtmann IS Lardies MA (1999) Egg production of *Austropandalus grayi* (Decapoda, Caridea, Pandalidae) from the Magellan region, South America. Scientia Marina 63: 325–331.

[B64] Welsh BL (1975) The role of grass shrimp *Palaemonetes pugio* in a tidal marsh ecosystem. Ecology 56: 513–530. doi: 10.2307/1935488

[B65] Wood E (2001) Global advances in conservation and management of marine ornamental resources. Aquarium Sciences and Conservation 3: 65–77. doi: 10.1023/A:1011391700880

